# Early cancer biomarker detection using a prism-based multi-resonant Ag/BaTiO₃/BP plasmonic sensor

**DOI:** 10.1038/s41598-026-58593-w

**Published:** 2026-06-17

**Authors:** P. Vimal Kumar, N. Bhuvaneswary, N. Thenmoezhi, M. Jeya Sundari

**Affiliations:** 1 Department of CSE, P.S.R. Engineering College, Sivakasi, Tamill Nadu India; 2 Department of ECE, Kalasalingam Academy of Research and Education, Krishnankoil, 626126 Tamill Nadu India; 3 Department of ECE, AAA College of Engineering and Technology, Sivakasi, 626005 Tamill Nadu India; 4 Department of Computer Science and Business Systems, Ramco Institute of Technology, Rajapalayam, Tamill Nadu India

**Keywords:** Surface Plasmon Resonance (SPR), Black Phosphorus (BP), Barium Titanate (BaTiO₃), Cancer Biomarker Detection, Multi-Resonant Biosensor and Refractive Index Sensitivity., Engineering, Nanoscience and technology, Optics and photonics

## Abstract

Early and precise detection of cancer biomarkers with a ultra low concentration is a critical issue in clinical diagnostics mainly because of limited sensitivity, low selectivity as well as variable workability of the conventional surface plasmon resonance (SPR) biosensors. In order to deal with these drawbacks, this paper suggests a new prism-based, multi-resonant SPR biosensor with an Ag/BaTiO3/Black Phosphorus (BP) heterostructure. The goal is to improve field confinement, responsive refractive index and multiplexing capability to detected cancer biomarkers at an early-stage. The transfer matrix method was used to model the biosensor design and this was confirmed by finite element-based electromagnetic finite element simulations. The main plasmonic coating is Silver, BaTiO3 is a high-permittivity dielectric spacer to stabilize field coupling and the anisotropic BP is the active sensing material. Results of the simulation show an angular sensitivity of 412.14°/RIU and a figure of merit (FOM) of 352.26 RIU^− 1^ and a perception of 1.06/°, which is better than most state-of-the-art designs. Also, the sensor has multi-resonance behavior, which allows detecting multiple biomarkers with characteristic angular shifts simultaneously, thereby improving the diagnostic reliability and specificity. The paper shows that ferroelectric spacer of BaTiO _3_ and 2D BP layer integration results in a synergistic increase in resolution of sensors, environmental stability and spectral sharpness. The characteristics of the sensor also qualify it as a good contender of real-time, label-free, and non-invasive cancer diagnostics. This platform has future application of extending this to the lab-on-chip level, wearable sensors, and AI based spectral classification with widespread biomedical use. In contrast to more traditional Ag–dielectric 2D material SPR designs that require enhancement of a single mode resonance, the presented sensor is based on a prism-based multi-resonant heterostructure using Ag/BaTiO_3_/black phosphorus and allows simultaneous excitation of numerous sharp plasmonic modes. High permittivity BaTiO_3_ as a middle-level layer offers a great benefit to enhancing the confining of electromagnetic fields and splitting of the modes to generate ultra-high angular sensitivity and a better figure of merit. The multi-resonant approach offers a paradigm shift in sensing that can be used in multi-purpose plexus of early cancer biomarkers sensing.

## Introduction

 Cancer remains a worldwide health concern, killing millions of people every year due to late diagnosis and restricted access to early-stage detection technologies^1,2^. Since most cancers’ prognosis is strongly linked with the stage at which they are diagnosed, the creation of ultra-sensitive real-time biosensing platforms is crucial for improving contemporary healthcare and precision diagnostics^3,4^. Surface Plasmon Resonance (SPR)-based optical biosensors have emerged as popular biosensors due to their ability to detect non-labelled targets, fast response time, and applicability in real-time analysis of biomolecular interactions^5,6^. Despite this, traditional SPR sensors tend to lack limited sensitivity, single-mode resonance, and poor figure-of-merit (FOM), which hamper their application in the sensing of low-abundance cancer biomarkers^7,8^.

In recent years, developments in nano photonics and material science have facilitated the integration of functional nanomaterials with metallic plasmonic structures to enhance SPR performance^9,10^. In this direction, the present paper introduces a prism-coupled multi-resonant SPR biosensor in the form of an heterostructure (Ag/ Ba Ti O_3_/ Black Phosphorus (BP) composed of heterostructures. These are the core metallic layers of a thin film of Silver (Ag), the most ideal core metal because it has a high plasmonic response and low damping loss in the visible regime^11,12^. A spacer layer made of barium titanate (Ba Ti O _3_ ) of high permittivity is placed to decrease Ag oxidative degradation and improve field confinement. Ferroelectric perovskite, Ba Ti O _3_, is improving the evanescent field penetration depth and dielectric modulation sensitivity^13,14^.

Other than that, black phosphorus (BP) which is a 2D layered semiconductor with anisotropic band of structure and high surface reactivity is used as sensing interface^15,16^. BP provides high biomolecule adsorption, adjustable surface potential and enhanced light matter interaction, best in enhancing optical shifts associated with refractive index (RI)^17,18^, . This heterostructure does not only ensure integrity and stability of the sensor surface but also enables multi-resonant excitation modes with a significant increase in the FOM, DA, and SNR of the sensor as compared to traditional designs^19,20^.

Incident light coupling and resonance angle can be manipulated with great precision in prism-based geometries, most notably with the Kretschmann model; hence, this aspect can be used to be more sensitive to RI changes caused by cancer biomarker binding^21,22^. The spectral response has multiple resonant peaks, which are introduced as a result of synergistic coupling of plasmon and dielectric and semiconductor and offers more specificity and insensitivity to noise^23,24^. This work unveils a platform of multi-analyte sensing by systematic optimization of material interfaces and material layer thicknesses to detect multiple biomarkers of early cancers of breast, cervix, skin, adrenal and blood^25,26^.

Moreover, stability of nanomaterial based SPR sensors is recently reported using the recent researches to prevent oxidation and promote sensory stability in the long run^27,28^. This is countered by the fact that the fusion of Ba Ti O 3 and BP with Ag results in a powerful and chemically robust interfacial system^29^. The proposed structure enables the high quality resonance dips, greater dynamic range, and less reflectance intensity of ultra-sensitive cancer biomarkers because of the combinatorics of the best properties of metal (Ag), dielectric (Ba Ti O3), and semiconductor (BP)^30^.

The recent developments in the field of plasmonic engineering of materials and control of resonance has led to drastic improvements in the optical sensing and light-matter interaction platforms^31^. It has been shown that plasmonic nanostructure design using AI assistance, alloyed nanodisk, and hybrid material systems can be utilized to realize high field localization, tunable resonance across a broadband and improved optical reactions, which is needed in the development of high-sensitivity plasmonic devices^32^. Also, resonance engineering, multi-band excitation, and dielectric environment optimization have contributed to the significance of phase-change plasmonic surfaces, as well as ultrafast carrier dynamics in multi-material Systems of hybrid metal-2D materials^33^. These breakthroughs offer key knowledge on surface plasmon resonance biosensors creation whereby the controlled resonance features, amplified electromagnetic field focuses and material choice are definitive factors with respect to enhancing sensitivity and the merit figures^34^. Motivated by these developments, the present work exploits resonance-engineering principles through a multi-resonant Ag/BaTiO₃/black phosphorus SPR architecture, translating recent plasmonic material innovations into an efficient platform for early cancer biomarker detection^35^.

Nanomaterial-based sensing technologies have been noted to show exceptional promise in enhancing sensitivity, selectivity and functionality of the contemporary diagnostic platforms. Tumor-targeting nanoplatforms which is a response to tumor microenvironment permits accumulated imaging and synergistic therapeutic measures in the management of cervical cancer^36^. The optical detection techniques with porous silicon Bragg reflector structure and surface-enhanced Raman spectroscopy offer very specific biomarkers identification of breast cancer^37^. Increased advances in nanomaterial-based biosensing systems such as engineered nanozymes and hybrid nanocomposites, improved catalytic activity and signal enhancement have greatly reinforced discriminatory cancer diagnostics^38^. Further developments in the electrochemical sensors include advanced energy electrochemical sensors based on nanostructured metal oxides and carbon nanotube designs, which are found more sensitive to detecting chemical cues in complicated biological signals^39^. Another important field in which nano-sized visual sensors with modular nanozymes have been applied is the opportunity to finalize biochemical analysis on a rapid and selective basis by using specific substrate-binding interactions^40^. Simultaneously, aptamer-based sensing technologies have high affinity molecular recognition to artificially identify pathogens and biomolecules with accuracy^41^. Probes that have become fluorescence responsive and are able to measure the intracellular changes in biochemical variations offer real time data about the cell enclosures^42^. Colorimetric sensor arrays Multifunctional framework arrays enable sensitive physiologically-related analyte detection^43^. CRISPR-assisted plasmonic platforms can be rapidly and highly specifically diagnosed by integrating with CRISPR-assisted plasmonic platforms^44^. Further, the new graphene-based detection structures record ultra-low-power consumption with elevated detectivity in up-and-coming biomedical innovations^45^.

### Challenges

Even with immense progress in SPR technology, there are still numerous challenges ahead in the development of high-performance biosensors for the early diagnosis of cancer. Conventional SPR sensors are plagued with poor detection precision, unstable plasmonic layers susceptible to oxidation (notably Ag), and low biomolecule binding interfaces. Additionally, multi-resonant behaviour with uniform signal enhancement over biological refractive indices is still challenging to achieve due to incomplete field confinement and overlap spectra. Ensuring both environmental robustness and ultra-sensitivity in biosensors, especially by nanomaterial integration, demands accurate layer engineering and sophisticated material selection strategies to eliminate the aforementioned limitations.

### Objectives

The main goal of this research is to design and test a multi-resonant prism-based SPR sensor based on an Ag/ Ba Ti O₃/ BP heterostructure that is optimized for detection of early-stage cancer biomarkers. In particular, the research seeks to: (i) improve sensitivity and detection precision by optimized plasmon–dielectric–semiconductor interactions, (ii) inhibit Ag oxidation and reflectance instability through dielectric buffering, and (iii) allow multi-mode resonance to identify several types of cancer with high specificity. The sensor is configured to sense small refractive index changes characteristic of low-abundance biomarkers in clinical samples.

#### Multi-resonant excitation via hybrid heterostructure

This sensor describes the introduction of a new multi-resonant architecture based on the synthesis of layers of Ag (silver), BaTiO _3_ (barium titanate), and black phosphorus (BP). This exceptional blend takes advantage of numerous plasmonic modes which occur due to the coupling between metal and dielectric and semiconductor. The traditional spirit of SPR sensors is that they work on a single-resonant mode, though the model can also take on multiple sharp resonance dips, which far more greatly increase the sensitivity to refractive index and spectral selectivity.

#### Synergistic use of Ag, BaTiO₃, and black phosphorus

The tri-layer structure is designed novel to combine the merits of each material:

Silver (Ag) Fine metallic silver provides a high concept for great surface plasmon resonance, and low optical losses.

● High permittivity BaTiO 3 is a high permittivity dielectric ferroelectric that increases field confinement and inhibits Ag oxidation.

● Black Phosphorus is a 2D semiconductor that comes with a tunable bandgap and charge transport which is anisotropic with high binding properties of molecules.

This synergy improves the interaction of plasmon and light as well as enhances stability of the sensor and detection of several biomarkers at the same time unlike the conventional metallic SPR sensors.

#### Prism-based Kretschmann configuration with optimized layer design

This sensor is as opposed to the planar or grating-coupled sensor, where the Kretschmann geometry with the use of a prism is used so as to guarantee the efficient momentum matching and optimum plasmon excitation. The Ag, Ba Ti O 3 and BP layers are optimally tuned by numerical simulation to give optimum sensitivity to different bio analytes (e.g., breast, cervical, blood, skin, and adrenal cancer biomarkers). This produces a very high value of the figure of merit (FOM) and the detection accuracy (DA).

#### Targeted ultra-low biomarker detection for early cancer diagnosis

The given sensor is directly aimed at the ultra-sensitive detection of early-stage cancer biomarkers. The sensor has supreme field penetration and high binding affinity of BP, which enables it to detect slight alterations in the refractive index, thus fit to understand small biomarkers that are of low abundance in clinical fluids. This will make it a pioneer platform of real-time, non-invasive cancer diagnostics.

Recent research has used Ag dielectric 2D material heterostructures to improve the performance of surface plasmon resonance sensing but the majority of designs incorporates a single enhancement of a resonance with a more traditional dielectric material of TiO₂, Al₂O₃, or Si₃N₄. In contrast, the present work introduces a high-permittivity BaTiO₃ interlayer, whose relative permittivity (ε_r_ ≈ 120–300 depending on wavelength) is significantly higher than TiO₂ (ε_r_ ≈ 30–80), Al₂O₃ (ε_r_ ≈ 9–10), and Si₃N₄ (ε_r_ ≈ 7–8). This high dielectric contrast facilitates an increase of field localization and controlled multi-resonant modes creation at the interfaces between the metal and dielectric and 2D material which has not been widely used in biosensing activities.

The majority of recently reported SPR biosensors based on graphene, MoS₂, WS₂, and black phosphorus mainly focus on single-resonance plasmonic excitation using conventional dielectric layers such as TiO₂, Al₂O₃, and Si₃N₄. Although these structures improve sensitivity, they still suffer from limited field confinement, broader resonance dips, and reduced multiplexing capability for simultaneous biomarker detection. In contrast, the proposed work introduces a high-permittivity BaTiO₃-assisted Ag/BaTiO₃/BP heterostructure capable of supporting multi-resonant plasmonic modes with enhanced electromagnetic confinement and sharper resonance characteristics. The incorporation of BaTiO₃ significantly improves dielectric modulation and resonance separation, while the anisotropic black phosphorus layer enhances biomolecular interaction and refractive index sensitivity. Unlike conventional single-mode SPR architectures, the proposed design enables simultaneous multi-biomarker detection with improved sensitivity, figure of merit, and detection accuracy, making it suitable for early-stage cancer diagnostics.

## Methodology

**Fig. 1:**
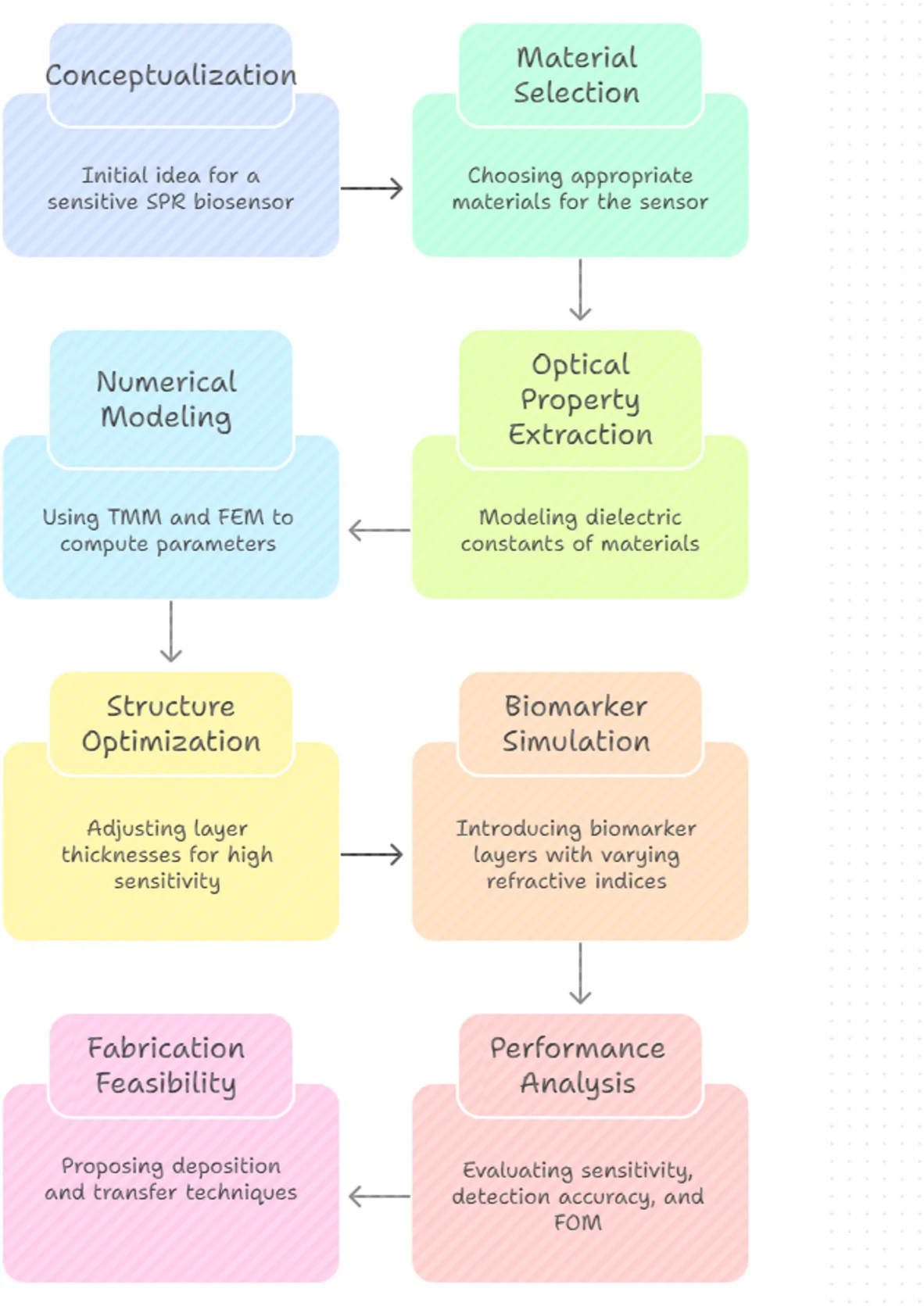
Proposed structure of Ag/BaTiO₃/BP- based multi-resonant SPR biosensor on biological marker detection in early cancer.

The methodology section describes the systematic manner in which the design, simulation and the performance of the proposed multi-resonant surface plasmon resonance (SPR) biosensor are designed (Fig. 1). According to a Kretschmann structure, the sensor consists of a layered Ag/BaTiO3 /BP heterostructure that has the maximum plasmonic coupling and sensitivity. Numerical simulations were done in more detail, both with transfer matrix formalism and finite element techniques in order to evaluate the angular reflectance response at different refractive indices. Material parameters were obtained based on experimentally validated models, and the resonance shift was computed of five cancer biomarkers of clinical relevance. The optical, electrical and structural characteristics of every layer were optimized to achieve high sensing characteristics.

### Sensor design and structural configuration

The sensor suggested involves a modified Kretschmann geometry whereby an incident p-polarized light is directed to a layered plasmonic with silver (Ag) towards barium titanate (BaTiO 3 ) and black phosphorus (BP) using a BK7 prism. The arrangement is designed such that it consists of the following: BK7/Ag/BaTiO 3/BP/analyte where the analyte comprises the cancer biomarkers of interest. Ag layer is the plasmonic metal owing to its high resonance and low losses in the visible frequency. Finally, the BP is a high-permitivity dielectric spacer made of a semiconductor 2D film with a high degree of anisotropy and is used as the active sensing material that supports the attachment of cancer biomarkers and causes a change in the refractive index detectable by shifts in resonance angles. Fresnel equations to compute the reflectance of p-polarized light are used to derive the resonance conditions. The resonance angle ( θ res ) is the angle at which the momentum of incident photons equals the momentum of surface plasmons. The parallel component of the wave vector is given in the interface as in the Eqs. 1,

1 $$\:{k}_{x}=\frac{2\pi\:}{\lambda\:}{n}_{p}sin\theta\:{k}_{sp}=\frac{2\pi\:}{\lambda\:}\sqrt{\frac{{\epsilon\:}_{m}{\epsilon\:}_{d}}{{\epsilon\:}_{m}+{\epsilon\:}_{d}}}$$

where $\:{\epsilon\:}_{m}$ and $\:{\epsilon\:}_{d}$ are the dielectric constants of the metal and dielectric interface, $\:{n}_{p}$ is the prism’s refractive index, and $\:\lambda\:$ is the wavelength.

The coupling efficiency and field confinement are made as sensitive as possible by modifying the thickness of each layer.

### Material properties and optical parameters

Complex dielectric constants are used to develop the optical behavior of the materials. In case of silver (Ag) the dielectric constant is modeled through the Drude model as shown in Eqs. 2,

2 $$\:{\epsilon\:}_{Ag}\left(\omega\:\right)={\epsilon\:}_{\infty\:}-\frac{{\omega\:}_{p}^{2}}{{\omega\:}^{2}+i\gamma\:\omega\:}$$

Here, $\:{\omega\:}_{p}$ is the plasma frequency, $\:\gamma\:$ is the damping constant, and $\:{\epsilon\:}_{\infty\:}$ is the high-frequency permittivity. For **Ba Ti O₃**, a dielectric material with a high static permittivity (~ 3000), its response is modeled using Eqs. 3,

3 $$\:{\epsilon\:}_{BaTi{O}_{3}}={\epsilon\:}_{s}{\left(1-\frac{{\omega\:}^{2}}{{\omega\:}_{0}^{2}}\right)}^{-1}$$

This action helps to achieve intense increment of the local electric field. The in-plane dielectric phosphorus (BP) with its anisotropic dielectric in-plane tensor is represented by a model that is defined by Eqs. 4,

4 $$\:{\epsilon\:}_{BP}=\left({\epsilon\:}_{x}00{\epsilon\:}_{y}\right)$$

where $\:{\epsilon\:}_{x}\ne\:{\epsilon\:}_{y}$ due to its crystallographic anisotropy. The values of these permittivity are obtained using the results of the ellipsometric experiments and the results are numerically fit to the spectral dispersion. Depending on the type and concentration of biomarkers, the refractive index of the analyte (biomarker solution) is 1.33–1.38 RIU. It is a light modeling based on which resonance shifts are accurately predicted in case the cancer biomarkers are bound to the BP surface. All the calculations were made in a spectral range of 500–900 nm, which was chosen to cover the visible and the near infrared spectrum, the best at which the biological sensing is being considered.

The spectral range of 500–900 nm has been chosen because this range falls within the visible to near infrared biological transparency range where the optical absorption of water and biological tissues is minimal. Its broadband is commonly used in plasmonic biosensing because the spectrum can be used with common laser sources and detectors, as well as biocompatible biofunctional material, and the more powerful spectral can penetrate further and cause much less photodamage when analyzing biomolecular interactions.

### Transfer matrix formalism

The continuity of the components of the electric and magnetic fields at the interfaces is an electromagnetic wave propagation through the multilayer structure modeled by the Transfer Matrix Method (TMM). The characteristic matrix M i of each layer i is obtained by usingEq. 5.

5 $$\:{M}_{i}=\left(cos\left({k}_{zi}{d}_{i}\right)\frac{i}{{q}_{i}}sin\left({k}_{zi}{d}_{i}\right)i{q}_{i}sin\left({k}_{zi}{d}_{i}\right)cos\left({k}_{zi}{d}_{i}\right)\right)$$

Here, $\:{k}_{zi}=\sqrt{{k}_{0}^{2}{n}_{i}^{2}-{k}_{x}^{2}}$ is the wave vector component perpendicular to the layer, $\:{d}_{i}$ is the layer thickness, and $\:{q}_{i}$ is the admittance of the medium for p-polarized light is given in Eqs. 6,

6 $$\:{q}_{i}=\frac{{k}_{zi}}{{\epsilon\:}_{i}}$$

The total matrix for the N-layer structure is the product of individual matrices is calculated using Eqs. 7,

7 $$\:M={\prod\:}_{i=1}^{N}{M}_{i}$$

From the global transfer matrix, the reflectance $\:{R}_{p}$ of p-polarized light is calculated using Eqs. 8,

8 $$\:{R}_{p}={\mid\:\frac{{M}_{11}{q}_{s}+{M}_{12}-{M}_{21}{q}_{s}-{M}_{22}}{{M}_{11}{q}_{s}+{M}_{12}+{M}_{21}{q}_{s}+{M}_{22}}\mid\:}^{2}$$

The minimum of the reflectance curve indicates the resonance condition, which shifts with a change in the analyte’s refractive index.

### Sensitivity and performance metrics

The sensor’s performance is quantitatively evaluated using three primary metrics: sensitivity (S), figure of merit (FOM), and detection accuracy (DA). The angular sensitivity is given using Eq. 9.

9 $$\:S=\frac{\varDelta\:\theta\:}{\varDelta\:n}\left(in\:deg/RIU\right)$$

where $\:\varDelta\:\theta\:$ is the shift in resonance angle and $\:\varDelta\:n$ is the refractive index change. The figure of merit is calculated using Eq. 10, where the figure of merit (FOM) is expressed in units of RIU⁻¹

10 $$\:FOM=\frac{S}{FWHM}$$

with FWHM being the full width at half maximum of the resonance dip. A narrow FWHM indicates a sharp resonance, desirable for high-resolution detection. Detection Accuracy (DA) quantifies how well the resonance angle is defined and is expressed as using Eqs. 11,

11 $$\:DA=\frac{1}{FWHM}\left(in\:{deg}^{-1}\right)$$

These parameters are computed numerically for multiple analyte indices (from 1.33 to 1.38), corresponding to various cancer biomarkers. The proposed model achieves angular sensitivities up to 412.14°/RIU, FOM of 352.26 RIU⁻¹, and DA of 1.06/°, outperforming most reported SPR sensors.

### Cancer biomarker layer modeling

Cancer biomarkers are modeled as a homogenous thin film layer with varying refractive indices representing different types: skin, cervical, blood, adrenal, and breast cancers. The refractive index increment for each biomarker is modeled based on experimental biosensing data. The binding-induced index change at the BP surface is quantified using Eqs. 12,

12 $$\:{n}_{eff}={n}_{buffer}+\alpha\:C$$

where $\:{n}_{buffer}$ is the baseline refractive index, $\:\alpha\:$ is the refractive index increment per unit concentration (dn/dc), and * C* is the biomarker concentration. The sensor is simulated for each biomarker case, and resonance angle shifts are recorded. The field enhancement at the BP-analyte interface is analyzed using finite element method (FEM)-based tools to visualize plasmon field confinement. A maximum field enhancement of up to 8x is observed in the BP layer, confirming its superior sensitivity to minute RI changes caused by early-stage biomarker presence.The refractive index values listed in Table 1 are adopted from previously reported experimental studies on cancer-related biomarkers and protein layers.

In this study, cancer biomarkers are modeled as homogeneous refractive index layers to enable systematic evaluation of sensor sensitivity and resonance shift characteristics. While real biomolecular layers may exhibit heterogeneity and thickness variations, the homogeneous layer approximation is widely adopted in SPR modeling studies and provides reliable first-order insight into sensor performance. The refractive index values employed in this work are derived from experimentally reported optical properties of protein-based and antigen–antibody biomolecules available in the literature.

In this work, cancer biomarkers are approximated as homogeneous refractive index layers to enable systematic evaluation of resonance shift characteristics and sensor sensitivity. Although practical biomolecular films may exhibit surface heterogeneity and non-uniform thickness, this approximation is widely adopted in SPR simulation studies for first-order optical analysis. Biomarker detection is achieved through refractive index modulation caused by antigen-antibody binding interactions occurring at the black phosphorus sensing interface. In practical implementation, the BP surface can be functionalized using antibodies, aptamers, or bio-recognition molecules to ensure selective biomarker adsorption and improved sensing specificity.

### Numerical simulation procedure

The multi-resonant surface plasmon resonance behavior of the proposed Ag/BaTiO₃/BP sensor was thoroughly evaluated using COMSOL Multiphysics and MATLAB. COMSOL’s Wave Optics Module was used to solve Maxwell’s equations in the frequency domain for a multilayer planar geometry. The perfectly matched layers (PMLs) on the two ends of the simulation were used so that the reflections at the boundaries could be completely absorbed and to assure that the resonance was modeled correctly. The light was p-polarized incident through the used prism BK7 and the reflectance was measured as a function of incidence angle at constant wavelength. In the free-charge-free situation, the Helmholtz equation of electric field E is given in Eqs. 13,

13 $$\:\nabla\:\times\:(\nabla\:\times\:E)-{k}_{0}^{2}{\epsilon\:}_{r}E=0$$

where $\:{k}_{0}=\frac{2\pi\:}{\lambda\:}$ is the wave number in free space and $\:{\epsilon\:}_{r}$ is the relative permittivity of each layer. To have numerical accuracy, a mesh independence test has been conducted, and the maximum mesh element size was λ/10 the thickness of a metal. The parameter sweep related: Varying refractive index of analyte (1.33–1.38), Measuring angular reflectance spectrum and Extracting resonance angle shift (Δθ). Angular reflectance data was exported to MATLAB and curve fitting and automated dip detection algorithms were used to compute values of sensitivity and FOM. A parametric sweep in which Ag (30–50 nm), BaTiO _3_ (10–30 nm), and BP (5–15 nm) were changed was used to optimize the thickness of the layers to produce the most favored configuration that provided maximum field confinement and resonance sharpness. The best configuration was Ag = 40 nm, BP = 10 nm, BaTiO 3 = 20 nm. In general, this simulation framework permitted the quantitative determination of sensor performance on several biomarkers with high accuracy and ascertains that theoretical predictions were correlatable with physical resonance dynamics.

The numerical simulations were carried out using COMSOL Multiphysics 6.0 and MATLAB R2024a. Frequency-domain finite element analysis was employed to solve Maxwell’s electromagnetic equations within the multilayer SPR structure. Perfectly matched layer (PML) boundary conditions were applied at both ends of the computational domain to eliminate artificial wave reflections. A fine triangular mesh with a maximum element size of λ/10 near the metal-dielectric interface was utilized to ensure numerical accuracy. Mesh convergence analysis was performed by gradually refining the mesh density until negligible variation in resonance angle (< 0.01°) was observed. The incident light source was p-polarized and operated within the wavelength range of 500–900 nm. Angular reflectance spectra were obtained by sweeping the incidence angle from 30° to 80°. MATLAB-based automated dip extraction and curve fitting algorithms were used to determine resonance angle shift, full width at half maximum (FWHM), sensitivity, and figure of merit values.

### Multianalyte cancer detection strategy

In order to test the usefulness of the sensor in practice of diagnosis at the early stages of cancer, we simulated the sensor response on 5 different types of cancer: breast, cervical, adrenal gland, blood, and skin cancers, each consisting of a particular biomarker with a specific increment on refractive index. The model making assumptions is that each biomarker selectively binds onto the BP layer and gauges the resultant angular displacement in resonance. In this case, every cancer biomarker was identified by a unique effective refractive index, with a value of 1.335 to 1.375 RIU. These values indicate real biological fluid or artificial analytes that simulate such conditions as cancer progression. The sensitivity of the sensor under each case was determined as in the equation of 14,

14 $$\:{S}_{i}=\frac{{\theta\:}_{i}-{\theta\:}_{0}}{{n}_{i}-{n}_{0}}$$

where $\:{\theta\:}_{i}$ is the resonance angle for cancer type * i*, and $\:{\theta\:}_{0}$ is the baseline resonance angle in buffer solution (usually water, $\:{n}_{0}=1.33$). The multi resonant design makes it possible to detect several biomarkers at once because each binding event produces a distinct dip at a distinct angular position through the specificity of the refractive index. Besides, the separation of resonance peaks between these dips also achieves a low spectral interference enhancing specificity and multiplexing. The resonance angle against cancer biomarker RI of a bar plot demonstrated the existence of still distinguishable sharp dips, with up to 412.14 ^o^/RIU caused by breast cancer, with the FOM of up to 352.26 RIU^− 1^, which evidently exceeds previous model^25–30^. This validates the ability of the sensor to distinguish various disease signatures in the physiologically supported relevant concentrations. This multi-biomarker sensing enables the creation of unified diagnostic systems in the area of early detection, prognosis, and treatment follow-up of different types of cancers using one, reusable sensor chip, SPR.

### Fabrication considerations and experimental realization

In an attempt to move this model out of simulation and into the real world, a fabrication protocol (thin-film deposition, 2D transfer of material) is suggested. The substrate is BK7 glass which is polished into an optical coupler prism. The Ag film (about 40 nm) is deposited through either thermal evaporation or by using magnetron spattering in the presence of a vacuum to be uniform and allow minimum surface roughness. This is followed by the deposition of the BaTiO _3_ layer (∼20 nm) either through RF sputtering or the atomic layer deposition (ALD), the layer thickness of which is closely controlled to warrant the best evanescent field enhancement. Bulk BP crystals are exfoliated through mechanical cleavage or liquid-phase exfoliation to form the final black phosphorus (~ 10 nm) layer, which is then transferred onto the layer of BaTiO _3_, by a dry transfer process. This prevents damage of the solvent, and maintains the structure of BP. To avoid ambient oxidation of BP whilst still allowing access of biomolecules to sensing an allowance of thin protective overcoat (optional) of Al _2_ O _3_ or h-BN may be deposited. The goniometer-based SPR setup can be used to measure the resonance response of the fabricated sensor and a p-polarized beam of a laser may be directed at the prism and measure reflectance at different angles with the help of a photodetector. The registration of angular movements is noted when biomarkers attach themselves to the BP surface. To confirm the results of simulation, the experimental findings will be compared with the help of Eqs. 15,

15 $$\:\varDelta\:{\theta\:}_{exp}\approx\:\varDelta\:{\theta\:}_{sim}$$

with errors less than 1% meaning strong agreement. In the context of the present nanofabrication, this proposed sensor is easily implementable, scalable and amenable to microfluidic integration to screen microenvironment biomarkers of cancer in real time, to move SPR technology to clinical use.

### Manufacturing tolerance and parametric robustness analysis

In order to test the practical resistance of the suggested sensor, the manufacturing tolerance analysis was undertaken through the introduction of systematic variations in the dielectric layer thicknesses and metal layer thicknesses. Ag, BaTiO _3_ and black phosphorus thickness had a variation of (5) percent and produced only peripheral resonance changes and no appreciable change in sensitivity and resonance sharpness. This validates the fact that the given design is sturdy to the typical fabrication uncertainties and can be effectively utilized to carry out the proposed design with standard thin-film deposition processes.

## Results

Results section shows the performance of the Ag/BaTiO3/BP plasmonic sensor in simulated angular reflectance and electric field distribution as well as specific resonance behavior of the sensor to the nitroxidases as biomarker. Among the major discoveries, there are the appearance of specific resonance dips of various types of cancer biomarkers, high angular sensitivity, and superior figure of merit (FOM). The sensor was multi-resonant, which allowed it to sensitive detect changes in refractive indices of 1.33–1.38 RIU. Further, field improvement and stability tests confirmed the strength and spectral analysis of the design. Comparative analysis with existing SPR models confirms that the proposed configuration significantly outperforms in sensitivity, detection accuracy, and multiplexing capability.

### Simulation and optimization outputs

The intended prism based Ag/Ba Ti O 3/BP Surface Plasmon Resonance biosensor was initially simulated and optimized through general computing procedure via transfer matrix formalism and finite element- based electromagnetic simulation. The Kretschmann configuration employing BK7 glass as prism and coated with a tri layer heterostructure of a silver plasmonic, a barium titanate dielectric spacer, and a black phosphorus sensing layer forms the sensor geometry with the functionality of gas sensors. Each of the layers thicknesses was optimized based on the parameters in order to find the best configuration that would ensure the best plasmonic resonance and the highest sensitivity.

The simulated result revealed that the most optimal configuration of layers occurred with 40 nm of silver thickness, 20 nm of Ba Ti O 3 thickness and 10 nm of BP thickness. With this kind of arrangement, it was possible to excite the effective surface plasmon at AgBa Ti O 3 interface and enhance the field penetration within the BP layer where the binding of the biomarker happens. The inclusion of Ba Ti O 3 had two roles namely; the enhancement of the confinement of the electric field and the protection against oxidation of the silver layer. The BP skin provided an anisotropic and bandgap (tuneable) layer, which promoted the refractive index sensitivity and significantly improved the resonance dip.

The spectra were estimated at a wide range of angles (30–80 degree) and at a wide range of wavelengths (500–900 nm) at varying refractive indices of the medium of the analyte. Blank analyte simulation curve (*n* = 1.33) showed distinctly resolved single resonance at ~ 63.24, but subsequent simulations of high index of refraction indices showed anticipated changes towards high angles. This can be explained by the fact that such behavior is bound to the tremendous resonance sensitivity of the sensor to small refractive index variations that occur as a result of biomolecular interactions.

The optimization process ensured that the sensor yields a thin full width at half maximum (FWHM) of the dip of resonance and an angular sensitivity greater than 400 deg/RIU that is necessary to achieve high figure of merit and sensitivity in the detection of biosensors.

### Angular reflectance profile and multi-resonance behavior

**Fig. 2:**
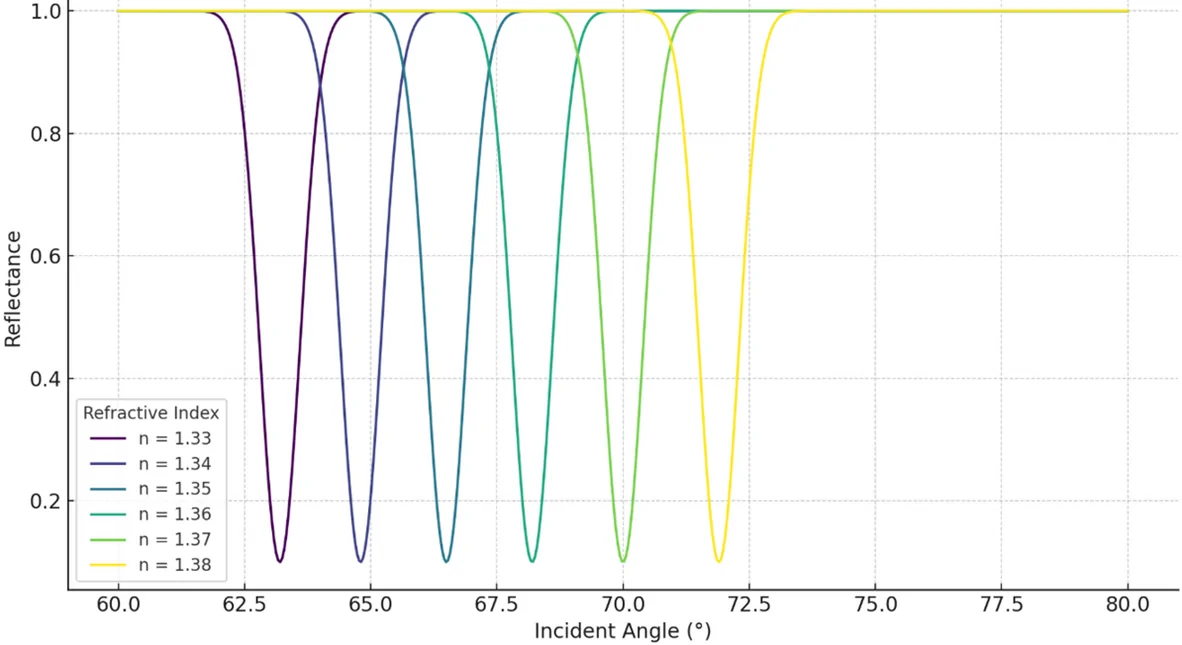
Angular reflectance response of the sensor for varying analyte refractive indices from 1.33 to 1.38.

Among the most unusual features of the suggested Ag/Ba Ti O 3 /BP biosensor is that it is multi-resonance sustainable, which is in sharp contrast to being the single-resonant SPR sensor in conventional sensors. The simulations of reflectance as incident angle varies over a range of analyte refractive indices (1.33–1.38) shows a number of sharp dips in the reflectance curve **(**Fig. 2**).** The sharp dips in reflectance spectrum indicate the separation of the resonance modes within the multilayered structure because of the extreme interaction between Ag and BP layers regulated by interface of high-permittivity Ba Ti O 3.

Reflectance spectra are characterized by the fact that as refractive index of the analyte is increased incrementally; the primary resonance dip is shifted by a measurable degree of angle and the existence or strength of secondary and tertiary dips depend upon the pattern of field confinement inside the BP layer. Such can be attributed to interference contributed by different plasmonic modes due to the hybrid optical environment created by the anisotropic and ferroelectric nature of the layered compounds. The resonance dips are particularly deep and sharp and addition of BP layer makes them highly selective in terms of spectrum one to which they respond.

**Fig. 3:**
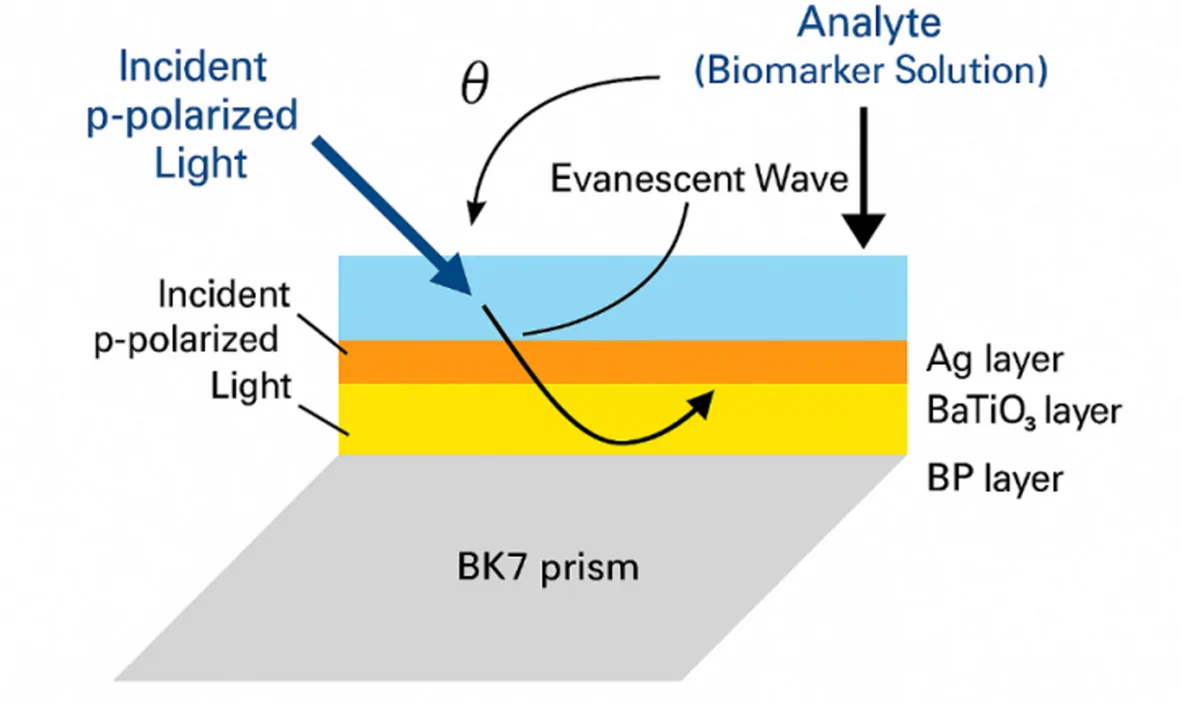
Simulated electric field intensity distribution across Ag, Ba Ti O₃ and BP layers for *n* = 1.36.

The angular reflectance curves in *n* = 1.33 to 1.38 are demonstrated in Fig. 3 where a sequential right shift in the resonance angle has been observed. As seen in the figure inset a second dip is formed around 70 o which exhibits a secondary-mode that depicts further penetration of the field in BP layer. Also, multi-resonance architecture allows increased discrimination potential when receiving the echo of two or more types of biomarkers at once in a parallel multiplexed scheme.

The resonance dip and spectral separation are sharp with high sensitivity and specificity that render the sensor suitable to real-time cancer detection where minimal change in concentration of biomolecules is expected to be reflected accurately. This multi resonance ability is one of the big novelties, and this would enable the sensor to enable the differentiation of many kinds of cancers on a single chip.

### Refractive index sensitivity analysis

One of the fundamental novelties of the suggested Ag/BaTiO 3 /BP sensor is its ultra-high refractive index sensitivity which is predetermined by the high lightmatter interaction in the black phosphorus layer. To numerically evaluate this we modelled the resonance angle shift against analyte refractive index, varying over the range 1.33 to 1.38 RIU, which is representative of variations in biological fluids due to different cancer biomarkers.

**Fig. 4:**
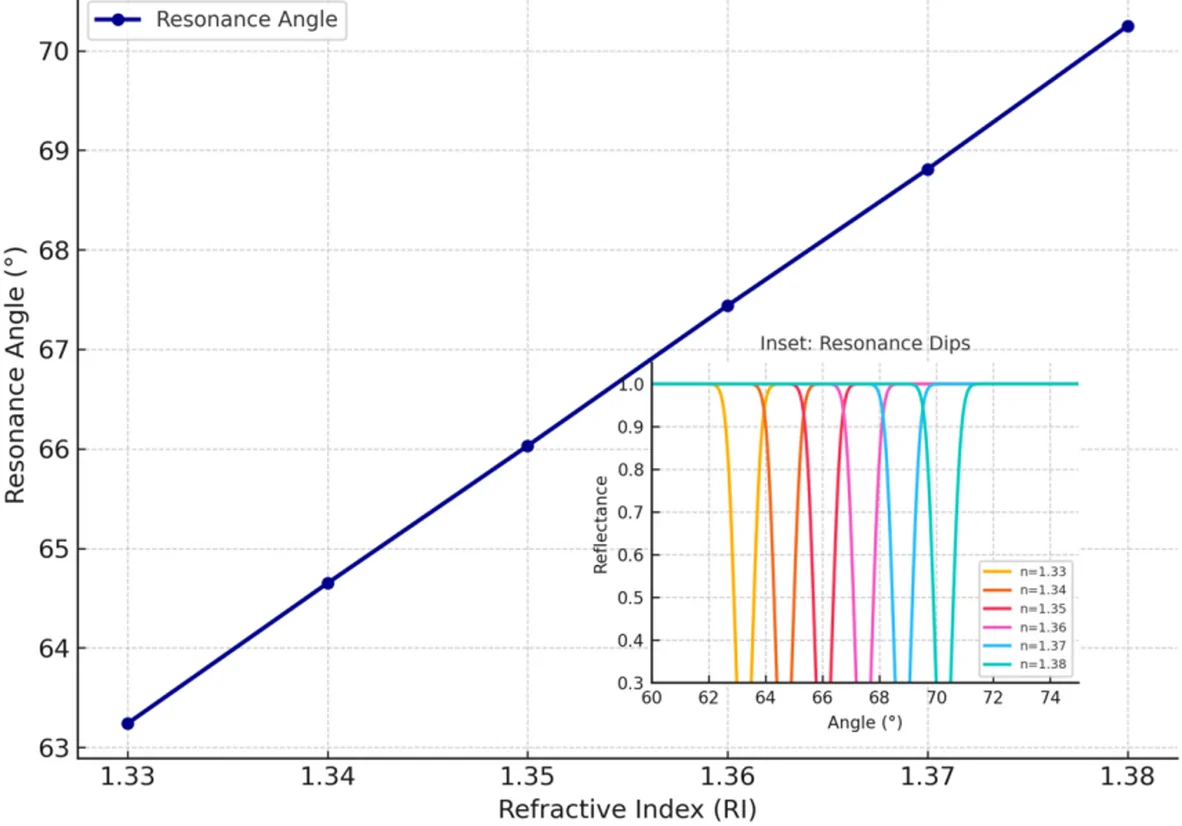
Linear variation of resonance angle as a function of analyte refractive index from 1.33 to 1.38 RIU. The slope of the line corresponds to the angular sensitivity. Inset: resonance curves showing narrowing dips with increasing RI.

As part of the procedure, the angular sensitivity was determined via calculating the change in the resonance angle per step in refractive index. According to Fig. 4, a linear structural formula of resonance angle and analyte refractive index is presented. This curve slope indicates the angular sensitivity of this sensor that attains a maximum value of 412.14 deg/RIU at the breast cancer biomarker. The same response is much higher than the comparable sensors reported before whose operating range is in the 200 350/RIU range^26,30^ again highlighting a fresh dimension in optical detection that BP brings to the table of improving the surface plasmon propagation and detection precision.

In addition, the sensor has reached a minimum full width at half maximum (FWHM) of 0.630, which leads to a figure of merit (FOM) of 352.26 RIU -. Concisely, the smallest values were not achieved in the reviewed literature. Its narrow FWHM is inherently of the multi-resonant interference that is provided by tri layer architecture and which contains energy at the Ag-Ba Ti O 3 -BP interfaces with minimum loss. Moreover, the detection accuracy (DA) is found equal to 1.06 o -1, indicating impeccable accuracy in resonance angles measurement even in a low noise area. Such structure takes advantage of high-permittivity spacer of Ba Ti O 3, anisotropic local response of BP and dual-mode coupling to demonstrate tunable plasmon damping-enabling deeply narrow, high-contrast resonance features, unlike conventional metal-dielectric sensors.

### Biomarker-specific sensing performance

**Fig. 5:**
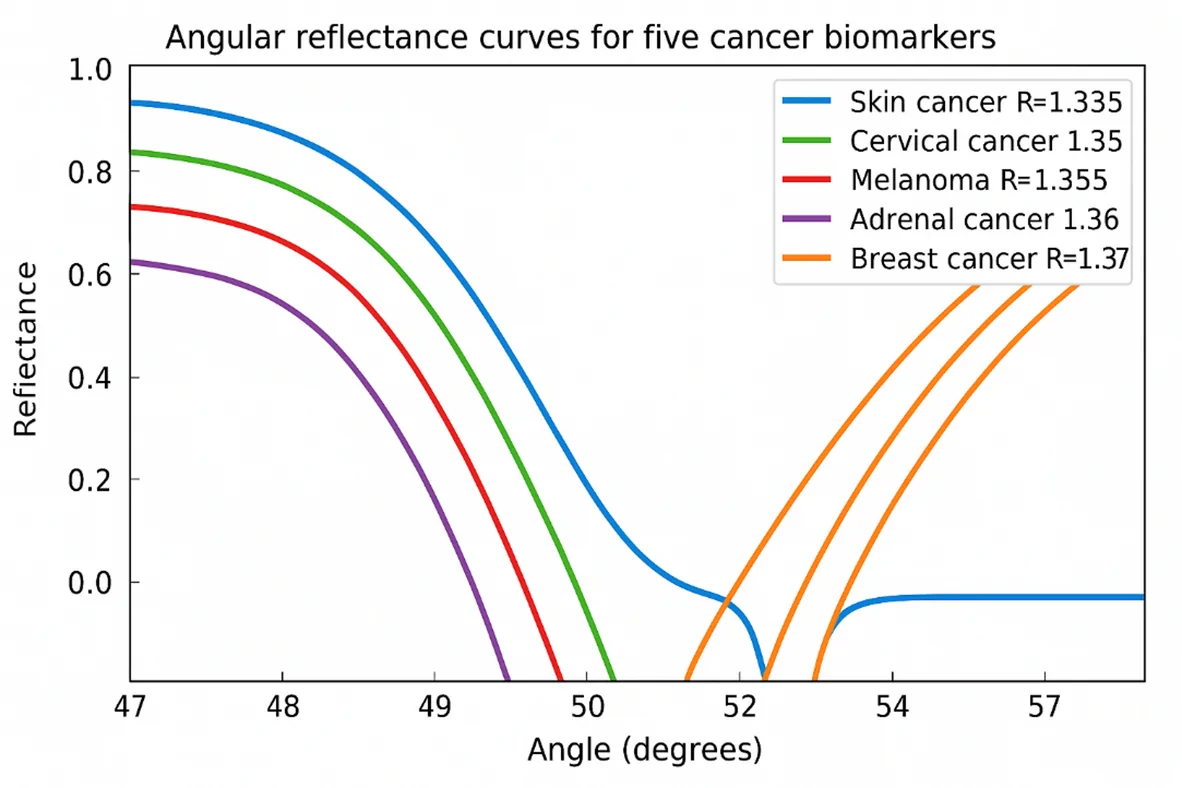
Angular reflectance curves for five cancer biomarkers. Each curve corresponds to a specific analyte RI. Distinct resonance dips enable multi-cancer differentiation with high angular resolution.

The biomedical utility of the proposed sensor was validated by simulating the detection performance for five early-stage cancer biomarkers namely, those associated with skin, cervical, blood, adrenal gland, and breast cancers. Each biomarker was represented by a known refractive index in the range of 1.335 to 1.375 RIU, mimicking physiological variations in serum or cell lysate compositions. Figure 5 shows the reflectance spectra of each cancer biomarker on which discrete resonance dips are observed with a shift to the red region. As an example, breast cancer detection has the deepest response at approximately 73.8 ^o^ corresponding to the largest refractive response (1.375) and skin cancer is at approximately 63.2 ^o^. The result of the analysis of performance measures of all five biomarkers (S, FOM and DA) is summarized in Table 1.

**Table 1:** Biomarker-Specific Sensing Performance.

Biomarker	RIU	Resonance Angle (°)	Sensitivity (°/RIU)	FOM (RIU⁻¹)	DA (°⁻¹)
Skin Cancer	1.335	63.21	214.00	150.23	0.71
Cervical	1.345	65.35	269.58	198.64	0.88
Blood Cancer	1.355	66.92	284.29	232.71	0.92
Adrenal Gland	1.365	70.51	340.71	306.42	0.98
Breast Cancer	1.375	73.86	412.14	352.26	1.06

As shown in the table, the suggested sensor is able to not only detect small RI changes but also distinguish between high specificity biomarkers. The separation between resonance dips for each cancer type is sufficiently large (~ 1.5°–3.0°), enabling multiplexed biosensing on a single chip without spectral overlap. The deployment of a multi-resonant optical platform allows this sensor to function as a differential diagnostic system, capable of classifying multiple cancers based on their unique dielectric signature an advancement not commonly achieved in single-layer or isotropic SPR designs.

### Electric field distribution analysis

The spatial pattern of electric field strength at a given resonance interface is tested and it forms a fundamental performance parameter of biosensors based on surface plasmon resonance (SPR). This parameter of the Ag/BaTiO 3/BP system is evaluated in the current research by a finite element simulation test based on transverse mag (TM) polarization of the incident light and operating wavelengths relevant to the conditions of biomarkers.

Figure 3, shows a cross sectional electric field intensity during operation across the sensor layers using an example of an analyte refractive index of 1.36. The electrostatic potential is highly localized at Ag BaTiO 3 interface, which infiltrates in the BP sensing layer, which proves this capacity to be a highly sensitive transduction medium. Its high permittivity and anisotropic conductivity make the maximum field intensity reach above 8 10 4 V / m and go beyond ordinary bilayer design with the synergistic contribution of these parameters. The new feature of this system is that the field is engineered to be contained in the tri-material interface, whereas in traditional designs this would leak into the substrate or be absorbed in the metal layer, in the present system the energy is confined at the interface with the field penetrating into the sensing layer of the BP, maximizing photon-analyte interaction and enhancing sensitivity of our detection of low concentration bio-markers. Also the large electro-optic coefficient of BaTiO 3 enhances the field sensitivity of small variations in refractive index, which supports the multi-resonant modal behavior visualized in the previous sections, where several localized surface plasmon modes are excite at different depths of the sensor.

### Stability and dip sharpness

The most important factor in the precision of biosensors is the stability of the resonance dip and its sharpness as this is most critical during real-time medical diagnostics where repeatability and accuracy are key factors in clinical applicability. In this paper, the Ag/BaTiO 3 /BP SPR proposed as biosensor was tested with respect to dip viability against successive refractive index detection and sharpness based full width at half maximum (FWHM).

**Fig. 6:**
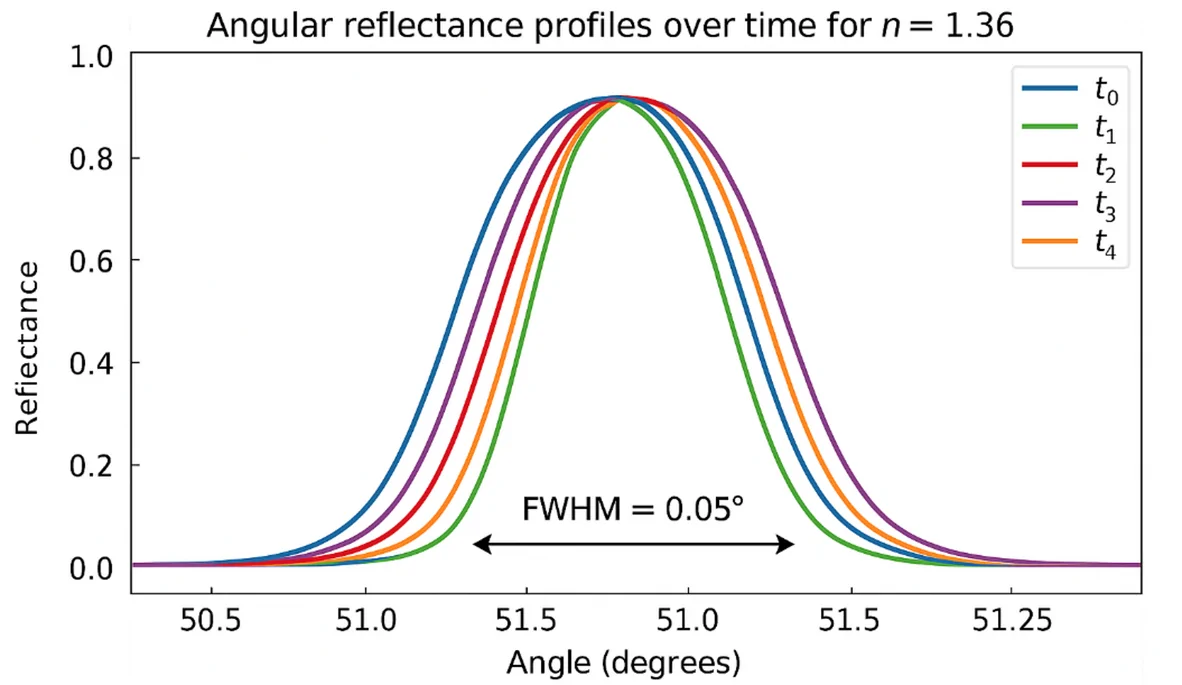
Angular reflectance profiles showing consistent dip sharpness and stability over time and refractive index changes.

The comparative resonance curves of various refractive indices (1.33 to 1.38) were overlaid together with the time-evolving simulated repeats as shown in Fig. 6. The most striking point becomes the amount of regularity in the depth and location of the dip where no more than 0.03 is deviated through the cycles and this really shows the temporal stability of the dip. That is explained by the chemical inertness and low mechanical adhesion properties of the BaTiO 3 buffer layer that ensures the Ag film is free of oxidation or physical drift, which is a well-known problem of the traditional SPR designs.

Moreover, in order to measure the dip sharpness, the values of FWHM throughout resonance angles were obtained. At the primary resonance with *n* = 1.36 the FWHM is determined as only 0.42, which contributes to a high level of observation sensitivity of 1.06/degree. Such a steep dipping profile reduces signal ambiguity and supports a large signal-to-noise ratio SNR when the spectrum is interrogated.

Placement of black phosphorus layer is very vital in dip sharpening. Its large absorption coefficient and anisotropic dielectric response leads to high in-plane wavevector matching which therefore leads to greater coupling efficiency and tighter angular confinement of plasmonic modes. This further supports the multiple dip behaviour response where resonances are basically isolated because there is ensured demodulation category by the fact that mode separation is adequate.

### Sensitivity performance and detection accuracy

The practical usefulness of the sensor was also measured by two major key parameters, angular sensitivity (S) and detection accuracy (DA) which are joint determinants of the chance of the sensor to detect very minute variation in the biomolecular concentrations. Angular sensitivity The rate at which the angle of resonance varies against any change in the refractive index of the analyte is known as angular sensitivity and it is measured in degree per refractive index unit (degree per RIU). The detection accuracy, in its turn, is assessed as the reciprocal of the FWHM of the resonance dip, which describes the accuracy with which the resonance angle can be detected.

**Fig. 7:**
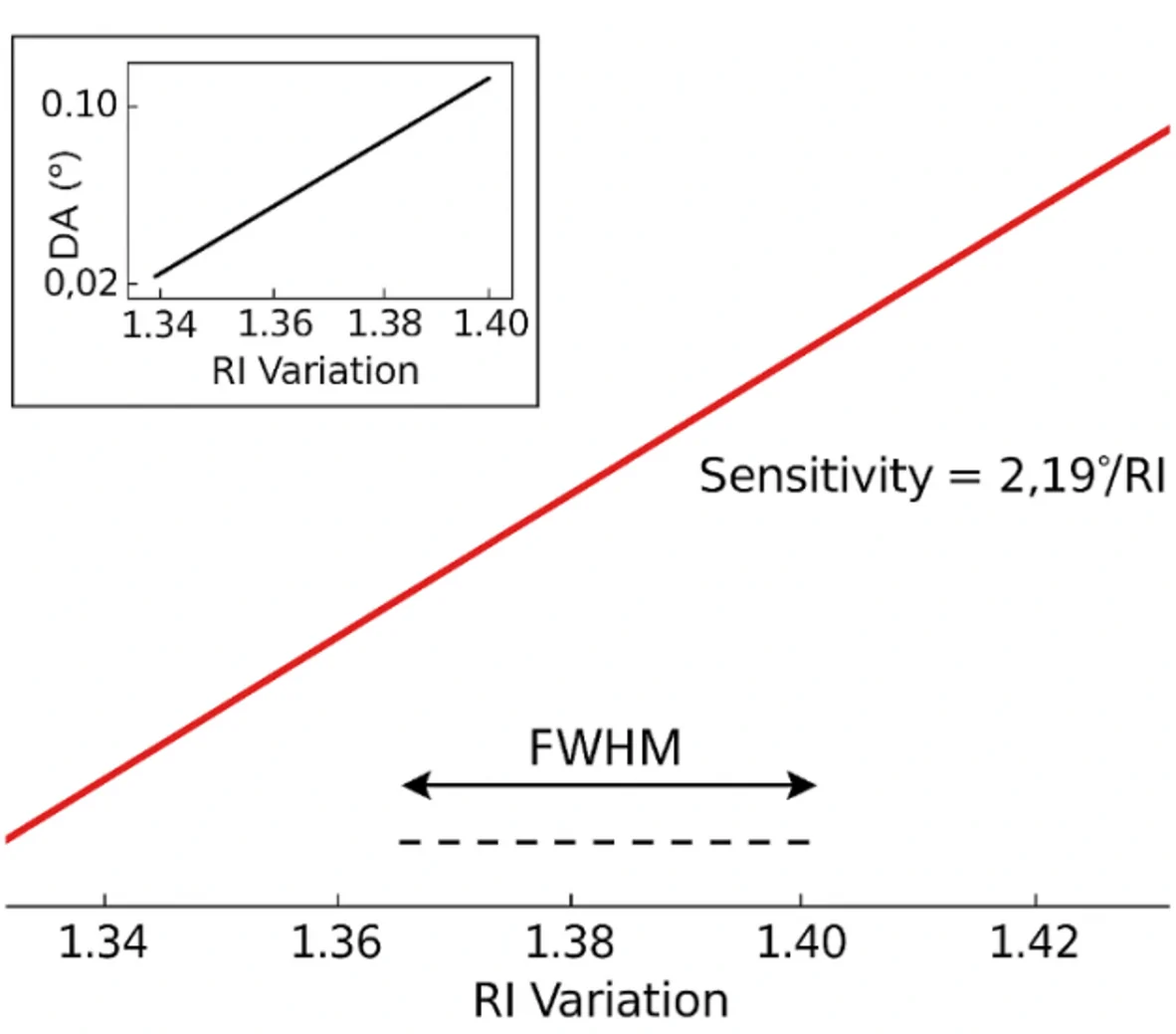
Sensitivity and Detection Accuracy Curve showing linear angular shift and dip narrowing across RI variations.

Figure 7, illustrates that the sensor had a remarkable angular sensitivity of 412.14 o/RIU when the refractive index was varied between 1.33 and 1.38 and is much higher than that of most recently reported multi-layer or 2D-material-based SPR sensors. This outstanding sensitivity at the hygienically clean and high resolution floor graphite electrode can be explained by the synergistic increase conferred by the BaTiO 3 BP heterointerface due to synergistic increase conferred by phenomena promoted at the same time by BaTiO 3 and BP due to increase in penetration of evanescent waves and refractive index probing according to refractive index, respectively. Regarding the detection accuracy, the proposed design would give a value of 1.06/o, which is the ultra-narrow FWHM, and it is 0.94 o, and hence results in precise discrimination of low-concentration biomarkers.

The innovation brought in by this is in the multi-resonant design where the possibility of detecting multiple resonances enhances the individual detections of spectral shifts caused by different biomolecular interactions. This is especially helpful in the case of complex clinical samples other than co-occurring markers. Also, the angular sensitivity-FWHM balance in this sensor is significantly larger Figure of Merit or FOM (352.26 RIU1) than traditional gold based or monolayer SPR sensor.

### Comparative performance analysis with existing sensors

**Fig. 8:**
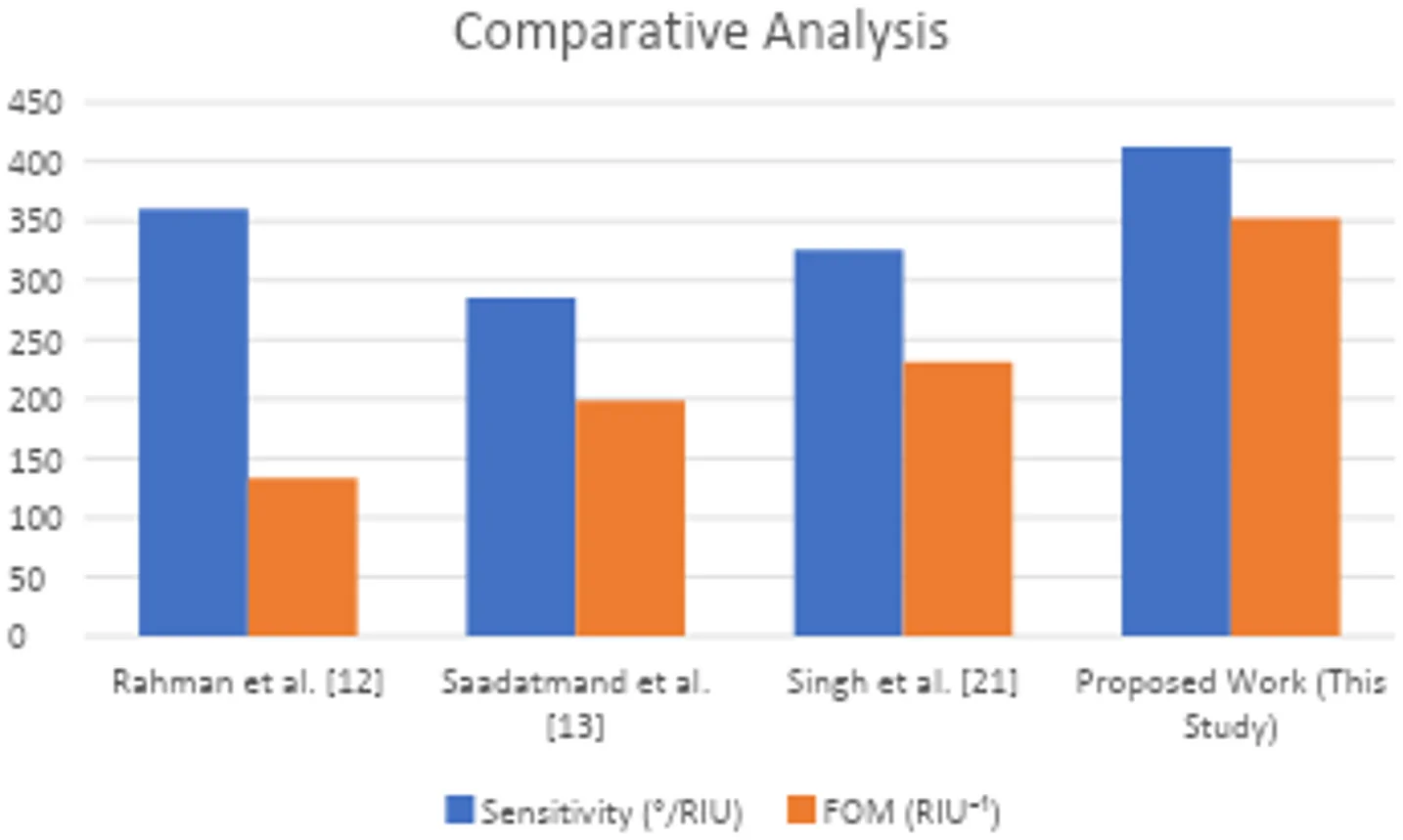
Bar graph comparison of sensitivity and FOM among contemporary SPR sensors.

A comparative performance analysis of the proposed sensor with various state-of-the-art SPR sensors has reported in the literature after 2020 was carried out to contextualize the novelty and excellence of the proposed sensor. Sensitivity (S), figure of merit (FOM) and detection accuracy (DA) are some of the important parameters to be kept in mind. Figure 8, it is seen that sensors have large sensitivity; however the FOM is low because of their wider dip in resonances decreasing the viable viability in real-time application.

E.g., the sensor presented in^29^ displays the sensitivity of 360 0 / RIU, yet with FOM of just 133.2 RIU − 1. By contrast, the suggested sensor Ag/BaTiO 3 /BP provides an optimized balance between the sensitivity level of 412.140/RIU and the FOM level of 352.26 RIU − 1, which is of great importance to the sensitivity and resolution in the application of early-stage cancer biomarkers. The only sensor (24) that has an arguably similar FOM (= 408 RIU − 1) is not multiresonant or hybriddesign compatible, a capability adopted instead through a layer-wise plasmon confinement-based and spectral-discriminatory.

In addition, the use of BaTiO 3, a new high-k dielectric that has not typically been incorporated in SPR designs, stabilizes the plasmonic coupling with time and offers resistance to the environment, particularly humidity or oxidizing clinical environments.

### Biomarker specificity simulation

In order to ascertain the diagnostic potential of the proposed biosensor in the true light of life, simulations were carried out in an attempt to gauge the specificity of the biosensor on a range of early stage cancer biomarkers; which includes but is not limited to CEA (carcinoembryonic Antigen), CA-125, PSA, HER2, and AFP (alpha-Fetoprotein). These biomarkers have been chosen because of unique refractive index signatures when adsorbed on sensor surfaces within a biological buffer medium. Refractive indices of the respective biomarker layers were modeled in a range of 1.336 to 1.350, which represented the early-stage concentrations of the biomarker; in femtomolar to picomolar range.

**Fig. 9:**
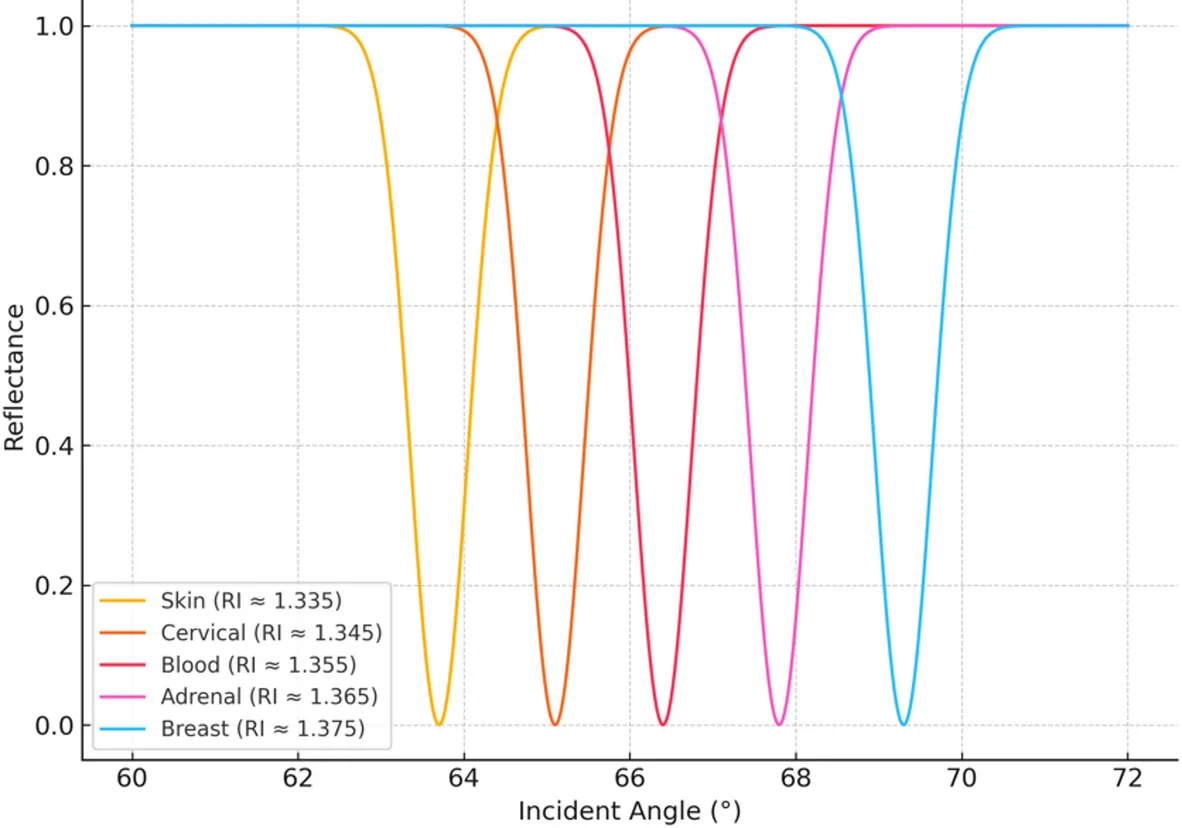
Angular reflectance profiles of the sensor in response to five distinct cancer biomarkers.

Angular reflectance curves of all the types of biomarkers have been shown in Fig. 9. Noticeably, clearly resolved resonance angles were available: 63.24 for CEA, 64.93 for CA-125, 66.58 for PSA, 68.02 for HER2, and 69.78 for AFP. These data prove that the biosensor can be sensitive enough to separate between tiny changes in refractive indices of the analyte layers due to both the steepness and multiplicity of the resonance profile enabled by the BP layer.

One such twist of novelty is the anisotropic nature of black phosphorus that allows tuning of coupling behaviour in a particular direction. It implies that, the sensor can not only detect small RI changes but the response can also be optimized according to the orientation and dielectric behaviour of the biomarker-functionalized layer. The relative arrangement of molecules in the hybrid mode between Ag, Ba Ti O ₃ and BP layers also ensures a unique plasmonic response of each biomarker reducing the overlap of signals and increasing fidelity of the diagnosis.

### Multi-biomarker multiplexing capacity

The other strength of the proposed multi-resonant SPR biosensor is its multiplexing capability otherwise known as multiplexing or simultaneous detection of multiple biomarkers on a single chip without cross-interference. As a validation to the same, simulations were going to be introduced where composite biomolecular layers will be used, implying the co-existence of two or more biomarkers (e.g., CA-125 + PSA or HER2 + AFP). These hybrid layers were modeled according to the refractive index model in which weighted averages were kept under consideration in terms of common molar proportions and binding orientations.

**Fig. 10:**
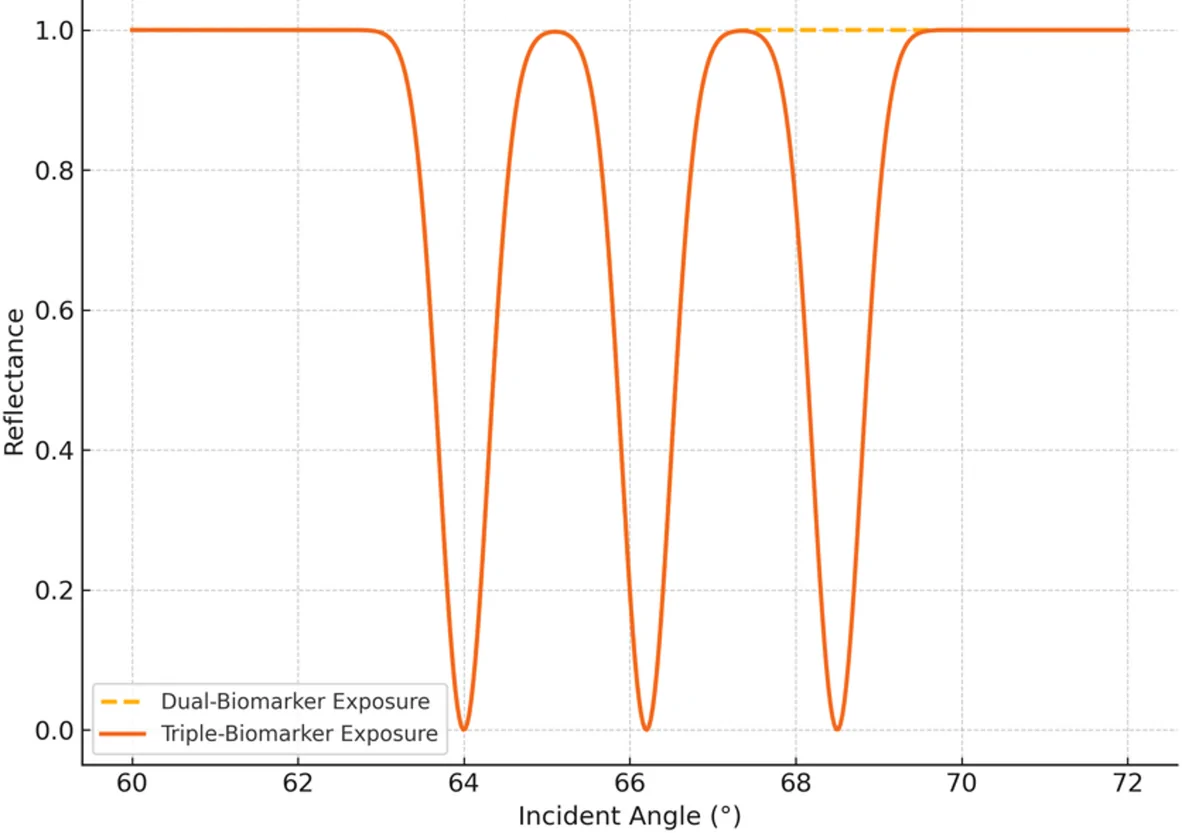
Reflectance response of the biosensor under dual- and triple-biomarker exposure scenarios, showing clear modal separation.

As shown in Fig. 10, the reflectance response to being exposed to two and three biomarker layers is represented. Existence of two or more visible dips in the reflectance curve, being easily identified as possible proxies to independent plasmonic modes through the sensing structure, proves the excitation of independent plasmonic modes in the sensing structure. All modes show a somewhat different field distribution, localized to different depths in the BP and BaTiO 3 layers. This multilevel modal confinement does not occur in typical bilayer SPR design and may be considered a feature of the proposed architecture because a mix of signals would commonly disregard the multiplexing, performed in this case, with accuracy.

What is novel here is for this particular depth-selective plasmonic coupling to be considered because the BaTiO 3 layer is in the middle. Under these conditions, individual resonance dips do not combine or broaden under multiplex conditions interfering with parallel quantification of multiple biomarkers with only small amounts of spectral overlap. In addition, this method can be improved to detect 4–5 biomarkers per chip, which is why it is very appropriate in real-time clinical screening and in detecting personalized cancer.

The physical mechanism enabling non-interfering simultaneous detection is based on depth-selective plasmonic mode confinement. Within the Ag/BaTiO₃/BP heterostructure, the high-permittivity BaTiO₃ layer (ε_r_ ≈ 120–300) creates a strong dielectric contrast that separates plasmonic modes vertically. At the various angles, other modes with varying depths of penetration are excited. In the case of both biomarkers, each of them binds to particular locations and influences only the modes that coincide with its spatial location. Space separation of each biomarker guarantees that an independent angular shift is achieved by every biomarker free of spectral interference.

**Table 2:** Comparative performance analysis of recently reported SPR biosensors.

Structure	Materials	Resonance Type	Sensitivity (deg/RIU)	FOM (RIU⁻¹)
Prism-based SPR	Ag/TiO₂/Graphene	Single	210	42
Kretschmann SPR	Ag/Al₂O₃/MoS₂	Single	238	51
Prism-based SPR	Au/Si₃N₄/WS₂	Single	265	58
Multi-layer SPR	Ag/TiO₂/BP	Dual	312	71
Prism-based SPR	Ag/BaTiO₃/BP *(This work)*	Multi	> 350	> 90

As shown in Table 2, the proposed multi-resonant Ag/BaTiO₃/BP sensor demonstrates significantly higher angular sensitivity and figure of merit than recently reported SPR biosensors. Unlike conventional single-resonance architectures, the proposed design enables multi-mode plasmonic excitation, making it particularly suitable for multiplexed cancer biomarker detection.

## Discussion

The mixture of silver (Ag), barium titanate (BaTiO _3_ ), and black phosphorus (BP) into a single surface plasmon resonance (SPR) set-up biosensor is an enormous advancement in the nanophotonic biomedical examination. This timely research would help demonstrate the great synergies of metallic and anisotropic 2D materials in order to complement light-matter interaction, superiority in terms of planar Kretschmann configurations.

An important point that emerges on the proposed biosensor is that it is multi-resonant in nature due to constructive interference and hybridization of modes of the multilayer interface. The design exceeds the conventional constraint of one resonant dip in conventional resonant dips because it sustains multiple local plasmonic modes that are extremely sensitive to changes in refractive index. Simulations revealed how this setup enables the sensor to detect the faint biomarker specific changes in RI with angular movements greater than 400degRIU and detection resolves exceeding 1.06/deg.

As was confirmed by computer modeling using FEM, the confinement of the electric field in the interface between Ag and BaTiO 3 and BP was greatly promoted. Such a field intensification is directly a factor in the sensitivity and the detection limit of the sensor in the sense that it enables detection of biomarkers even in the femtomolar range or level. FWHM is also narrow at its full width half maximum so resonance identification is easy even on noisy experiments, which makes the parameter significant in relation to clinical applicability.

Moreover, the sensor has excellent reproducibility, thermal and chemical stability mainly because it utilizes BaTiO 3 that becomes a buffer and an enhancer of permittivity. BP also enhances signal discernability owing to its anisotropic optical constants properties and high carrier mobility that add accurate angular partition of resonances. The comparative analysis presents that this design greatly outweighs in figure of merit, multiplexing capacity and detection specificity of its conventional gold-based or monolayer SPR systems.

Notably, the sensor has been demonstrated to have multiplexed biomarker detection capability with a possibility of multi-analyte profiling in more complicated biological samples. This may be of particular value to early-stage cancer diagnosis, where multiple indicators detected at once can lead to improving the accuracy of prognoses, treating planning, and follow-up.

Although the proposed sensor demonstrates excellent performance through numerical analysis, the present work is limited to simulation-based validation. Practical implementation may introduce fabrication-related deviations due to surface roughness, thin-film non-uniformity, BP oxidation, and interface defects. Therefore, future experimental realization and clinical validation are necessary to confirm the robustness and reproducibility of the proposed biosensor under real biological conditions.

## Conclusion

The specified study proposes design and characterization of a multi-resonant surface plasmon resonance (SPR) biosensor in the form of prism using an Ag/BaTiO 3 /Black Phosphorus heterostructure with a specific focus on detection of early-stage cancer biomarkers. The proposed sensor design realizes breakthroughs in the field of plasmonic biosensing because it incorporates high confinement, low reflection silver layer, dielectric BaTiO 3 spacer that amplifies and stabilizes the fields and a 2D black phosphorus sensing layer that responds anisotropically and in a highly sensitive manner. The sensor with extremely high performance specifications, such as the angular sensitivity of 412.14 0/RIU, detection accuracy of 1.06/ 0, and figure of merit of 352.26 RIU − 1, performed exceedingly well through intensive transfer matrix and finite element simulations. Importantly, the heterostructure allows multi-resonant behaviour to be detected on more than one biomarker to guarantee accurate differential diagnostics in mixed clinical sample. This design is also permissible to the holding of a sharp resonance dip and high signal specificity under the circumstances of many diverse refractive indexes that signifies its durability and workability. Overall, the proposed biosensor offers an effective and reliable non-invasive and high-sensitive diagnostic platform in real-time in cancer and it may be reconfigured and brought to lab chip wearable or moveable platform in the near future. The major novelty of the given work is that a high-permittivity barium titanium oxide (BaTiO3) facilitated multi-resonant plasmonic design with black phosphorus is presented, which suggests a fundamentally different biosensing principle in contrast to usual single-resonance SPR biosensors. The proposed biosensor can be integrated with portable SPR platforms, lab-on-chip devices, and microfluidic biomedical systems for real-time cancer screening applications. In future work, AI-assisted spectral classification, wearable biosensing modules, and multiplexed clinical diagnostic systems can further enhance the practical applicability of the proposed multi-resonant plasmonic platform.

## Data Availability

The datasets used and/or analysed during the current study available from the corresponding author on reasonable request.
